# Genomic Sequencing of Ranavirus Isolates from a Three-Spined Stickleback (*Gasterosteus aculeatus*) and a Red-Legged Frog (*Rana aurora*)

**DOI:** 10.1128/MRA.00902-21

**Published:** 2021-11-18

**Authors:** Cody R. K. Conrad, Kuttichantran Subramaniam, V. Gregory Chinchar, Thomas B. Waltzek

**Affiliations:** a Department of Infectious Diseases and Immunology, College of Veterinary Medicine, University of Florida, Gainesville, Florida, USA; b Department of Microbiology and Immunology, University of Mississippi Medical Center, Jackson, Mississippi, USA; KU Leuven

## Abstract

Two ranavirus isolates were recovered during a wildlife disease investigation in California in 1996. Preliminary testing at the time of sample collection indicated that the two isolates were identical. Phylogenetic analysis of the full genomes of these two isolates revealed that they are a single strain of frog virus 3.

## ANNOUNCEMENT

*Frog virus 3* (FV3), the type species of the genus *Ranavirus*, is capable of infecting fish, amphibians, and reptiles (1). FV3 possesses a linear, terminally redundant, double-stranded DNA genome, approximately 106 kb in length, that is packaged within an icosahedral nucleocapsid (2, 3). Two ranavirus strains, i.e., stickleback virus (SBV) and tadpole virus 2 (TV2), were isolated from tissue pools of three-spined sticklebacks (*Gasterosteus aculeatus*) and northern red-legged frogs (*Rana aurora*), respectively, during a wildlife disease investigation in Redwood National Park, California, in 1996 (4). Reddening of the skin, lethargy, and disorientation were observed in some of the three-spined sticklebacks, while no clinical signs were observed in the northern red-legged frogs. Ranavirus infection was confirmed based on protein profile analysis of SBV and TV2 cell culture isolates and comparison of their partial major capsid protein nucleotide sequences to that of FV3. Furthermore, restriction fragment length polymorphism (RFLP) analysis indicated that SBV and TV2 were markedly similar to FV3 (4).

The SBV and TV2 isolates were maintained for more than 20 years at –80°C until propagated in *Epithelium papillosum cyprini* cells as described previously (5). Cell culture supernatants were harvested at the third passage, and the total DNA was purified using a DNeasy blood and tissue kit (Qiagen) according to the manufacturer’s instructions. DNA sequencing libraries were multiplexed and constructed from the DNA extracts using a Nextera XT DNA library preparation kit (Illumina) according to the manufacturer’s instructions. The genome sequencing was performed using a v3 chemistry 600-cycle kit on a MiSeq sequencer (Illumina). Next-generation sequencing generated 3,313,540 reads and 3,070,856 reads for SBV and TV2, respectively. *De novo* assembly of the paired-end reads in SPAdes v3.12.0 (6), using default parameters, produced contiguous consensus sequences of 105,673 bp and 105,671 bp, with G+C contents of 55% each, for SBV and TV2, respectively. SBV and TV2 were identical except for a two-nucleotide (CA) addition in a repeat region in the former. Variations within repeat regions have been observed among Ambystoma tigrinum virus isolates (7).

**FIG 1:**
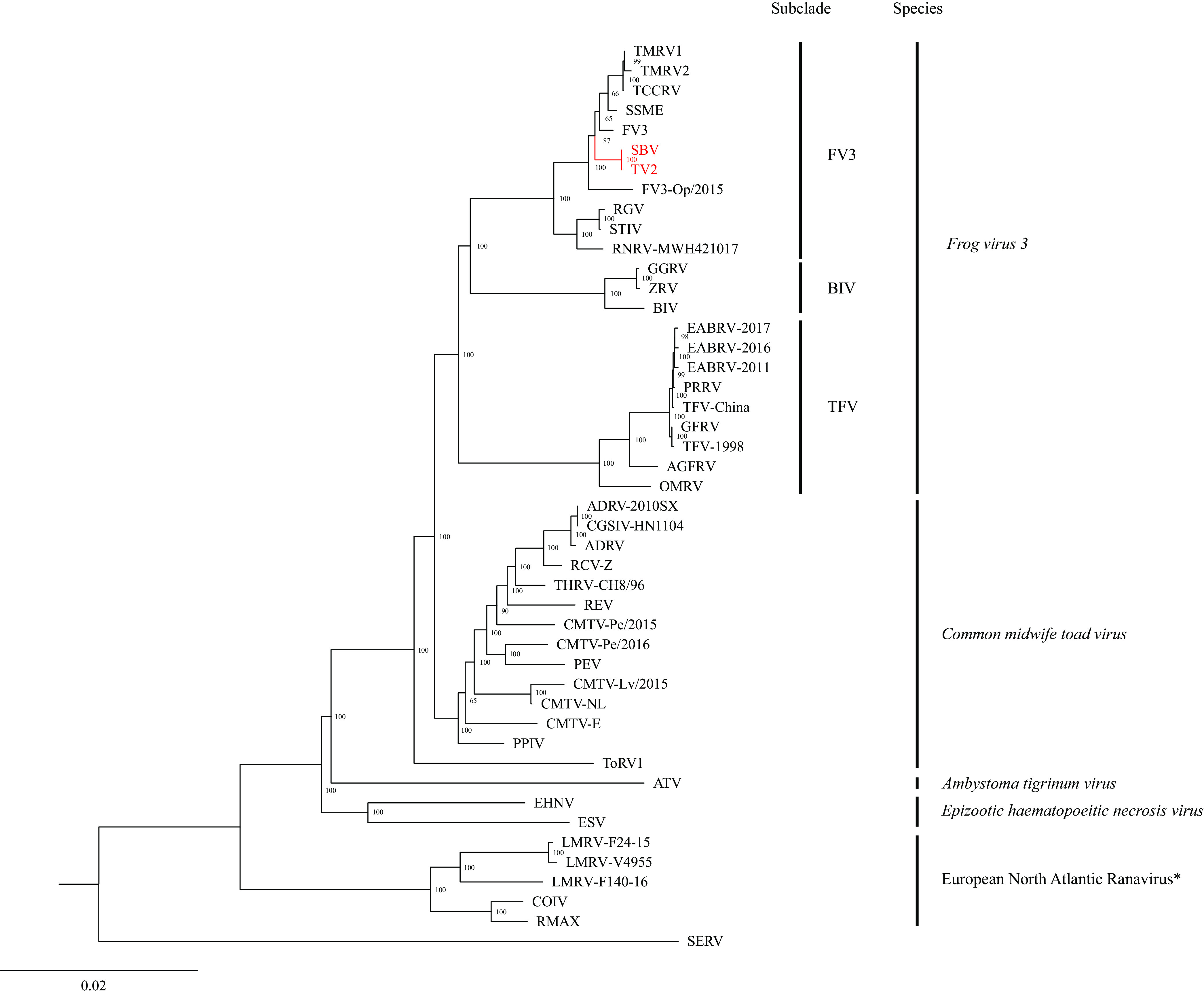
ML phylogram depicting the relationships of SBV and TV2 (in red) to 44 ranaviruses, based on the concatenated genome-wide LCB alignments. ML analysis was performed in IQ-TREE (http://iqtree.cibiv.univie.ac.at) with default parameters and 1,000 bootstrap replicates. The bootstrap values are provided at each node. See Table 1 for virus abbreviations. *, European North Atlantic ranavirus has not been approved as a ranavirus species by the International Committee on Taxonomy of Viruses.

The Genome Annotation Transfer Utility was used to annotate the full genomes of the two isolates, with Terrapene carolina carolina ranavirus (GenBank accession number MG953518) as the reference genome (https://4virology.net/virology-ca-tools/gatu/). Ninety-five putative open reading frames (ORFs) were predicted for both SBV and TV2. The genome-wide locally colinear block (LCB) alignments of 44 fully sequenced ranaviruses were generated in Mauve v2.4 with default parameters (Table 1). The LCB alignments were then concatenated in Geneious v7 (8) and used in a maximum likelihood (ML) analysis in IQ-TREE (http://iqtree.cibiv.univie.ac.at) with default parameters and 1,000 bootstrap replicates. The resulting ML tree supported SBV and TV2 as FV3 strains (Fig. 1).

**TABLE 1:** Virus names, abbreviations, and GenBank accession numbers for the ranaviruses used in the phylogenetic analyses

Virus abbreviation	Virus name	GenBank accession no.
FV3	Frog virus 3	AY548484
TFV-China	Tiger frog virus	AF389451
TFV-1998	Tiger frog virus isolate AV9803	MT512504
GFRV	Tiger frog virus isolate F0207	MT512501
RGV	Rana grylio iridovirus	JQ654586
EABRV-2011	Tiger frog virus isolate D11-067	MT512498
EABRV-2016	Tiger frog virus isolate VD-16-006	MT512499
EABRV-2017	Tiger frog virus isolate VD-17-007	MT512500
AGFRV	Tiger frog virus isolate D03-034	MT512497
OMRV	Tiger frog virus isolate D2008	MT512502
STIV	Soft-shelled turtle iridovirus	EU627010
BIV	Bohle iridovirus	KX185156
GGRV	German gecko ranavirus	KP266742
ATV	Ambystoma tigrinum virus	AY150217
EHNV	Epizootic hematopoietic necrosis virus	FJ433873
ESV	European sheatfish virus	JQ724856
CMTV-E	Common midwife toad virus	JQ231222
CMTV-NL	Common midwife toad virus	KP056312
THRV-CH8/96	Testudo hermanni ranavirus	KP266741
ToRV1	Tortoise ranavirus isolate 1	KP266743
SSME	Frog virus 3 isolate SSME	KJ175144
ADRV	Andrias davidianus ranavirus	KC865735
SERV	Short-finned eel ranavirus	KX353311
RMAX	Ranavirus maximus	KX574343
COIV	Cod iridovirus	KX574342
PPIV	Pike-perch iridovirus	KX574341
LMRV-F140-16	Lumpfish ranavirus isolate F140-16	MH665359
LMRV-F24-15	Lumpfish ranavirus isolate F24-15	MH665358
LMRV-V4955	Lumpfish ranavirus isolate V4955	MH665360
ADRV-2010SX	Andrias davidianus ranavirus	KF033124
CGSIV-HN1104	Chinese giant salamander iridovirus	KF512820
CMTV-Lv/2015	Common midwife toad virus	MF004272
CMTV-Pe/2015	Common midwife toad virus	MF125269
CMTV-Pe/2016	Common midwife toad virus	MF125270
PEV	Pelophylax esculentus virus	MF538627
PRRV	Tiger frog virus isolate F2112	MT512503
SBV	Stickleback virus isolate 1096	MZ514903
TV2	Tadpole virus 2	MZ514904
RCV-Z	Rana catesbeiana virus isolate RC-Z	MF187210
REV	Rana esculenta virus	MF538628
TMRV1	Trioceros melleri ranavirus 1	MG953519
TMRV2	Trioceros melleri ranavirus 2	MG953520
TCCRV	Terrapene carolina carolina ranavirus	MG953518
FV3-Op/2015	Frog virus 3 isolate Op/2015/Netherlands/UU3150324001	MF360246
RNRV-MWH421017	Rana nigromaculata ranavirus isolate MWH421017	MG791866
ZRV	Zoo ranavirus isolate 040414	MK227779

This study confirms that the SBV and TV2 strains isolated from two vertebrate species of distinct classes in a wild setting are nearly identical (4). The potential impact of FV3 is far-reaching, as strains are capable of infecting hosts of different vertebrate classes and can be found in animals of ecological and commercial importance (9).

### Data availability.

The complete genome sequences of SBV and TV2 have been deposited in GenBank under accession numbers MZ514903 and MZ514904, respectively. Raw sequence data for SBV and TV2 have been deposited in the Sequence Read Archive (SRA) database under accession numbers SRR15339644 and SRR15339645, respectively.
